# Insulin-induced gene 2 protects against hepatic ischemia–reperfusion injury via metabolic remodeling

**DOI:** 10.1186/s12967-023-04564-y

**Published:** 2023-10-19

**Authors:** Yichao Wu, Changbiao Li, Abid Ali Khan, Kangchen Chen, Renyi Su, Shengjun Xu, Yiyang Sun, Fengqiang Gao, Kai Wang, Xiaodong Wang, Zhengxing Lian, Shuo Wang, Mengyuan Yu, Xin Hu, Fan Yang, Shusen Zheng, Nasha Qiu, Zhikun Liu, Xiao Xu

**Affiliations:** 1 Zhejiang University School of Medicine, Hangzhou, 310058 China; 2 Key Laboratory of Integrated Oncology and Intelligent Medicine of Zhejiang Province, Hangzhou, 310006 China; 3 NHC Key Laboratory of Combined Multi-Organ Transplantation, Hangzhou, 310003 China; 4 Department of Hepatobiliary and Pancreatic Surgery, Shulan (Hangzhou) Hospital, Hangzhou, 311112 China; 5 The Fourth School of Clinical Medicine, Zhejiang Chinese Medical University, Hangzhou, 310053 China

**Keywords:** Insulin-induced gene 2, Hepatic ischemia–reperfusion injury, Pentose phosphate pathway, Glycolysis

## Abstract

**Background:**

Hepatic ischemia–reperfusion (IR) injury is the primary reason for complications following hepatectomy and liver transplantation (LT). Insulin-induced gene 2 (Insig2) is one of several proteins that anchor the reticulum in the cytoplasm and is essential for metabolism and inflammatory responses. However, its function in IR injury remains ambiguous.

**Methods:**

Insig2 global knock-out (KO) mice and mice with adeno-associated-virus8 (AAV8)-delivered Insig2 hepatocyte-specific overexpression were subjected to a 70% hepatic IR model. Liver injury was assessed by monitoring hepatic histology, inflammatory responses, and apoptosis. Hypoxia/reoxygenation stimulation (H/R) of primary hepatocytes and hypoxia model induced by cobalt chloride (CoCl_2_) were used for in vitro experiments. Multi-omics analysis of transcriptomics, proteomics, and metabolomics was used to investigate the molecular mechanisms underlying Insig2.

**Results:**

Hepatic Insig2 expression was significantly reduced in clinical samples undergoing LT and the mouse IR model. Our findings showed that Insig2 depletion significantly aggravated IR-induced hepatic inflammation, cell death and injury, whereas Insig2 overexpression caused the opposite phenotypes. The results of in vitro H/R experiments were consistent with those in vivo. Mechanistically, multi-omics analysis revealed that Insig2 is associated with increased antioxidant pentose phosphate pathway (PPP) activity. The inhibition of glucose-6-phosphate-dehydrogenase (G6PD), a rate-limiting enzyme of PPP, rescued the protective effect of Insig2 overexpression, exacerbating liver injury. Finally, our findings indicated that mouse IR injury could be attenuated by developing a nanoparticle delivery system that enables liver-targeted delivery of substrate of PPP (glucose 6-phosphate).

**Conclusions:**

Insig2 has a protective function in liver IR by upregulating the PPP activity and remodeling glucose metabolism. The supplementary glucose 6-phosphate (G6P) salt may serve as a viable therapeutic target for alleviating hepatic IR.

**Supplementary Information:**

The online version contains supplementary material available at 10.1186/s12967-023-04564-y.

## Introduction

Hepatic ischemia–reperfusion (IR) injury occurs when the restoration of blood flow follows prolonged ischemia. It is a potentially serious complication during liver transplantation (LT), liver resection, and hypovolemic shock [1, 2]. The IR process has been recognized as a detrimental event not just for postoperative graft dysfunction but also for acute and chronic rejections of grafts [3]. The pathogenesis of IR injury in the liver involves a cytotoxic cascade with high reactive oxygen species (ROS) production and damage-associated molecular patterns (DAMPs) leakage, leading to redox imbalance, inflammation, and cell death in injured hepatocytes. Hepatocytes are the pivotal parenchymal cells vulnerable to metabolic disturbances during the ischemia phase, triggering early necrosis; in the reperfusion phase, activated immune cells release inflammatory cytokines into liver tissues, thus aggravating cellular damage [4, 5]. Despite the proposal of various methods to improve liver damage induced by IR, including ischemic preconditioning and pharmacological interventions [6–8], the underlying pathways responsible for liver IR injury remain uncertain. Currently, there is a lack of clinically effective strategies for preventing or treating liver IR. Thus, elucidating the mechanism of IR is imperative for developing targets for treatment and relieving the shortage of donors for life-saving LT.

The endoplasmic reticulum (ER)-anchored protein, insulin-induced gene (Insig), retains the sterol regulatory element-binding protein (SREBP)-SREBP cleavage-activating protein (SCAP) complex in the ER, negatively affecting SREBP activation and lipogenesis in the liver [9]. Two Insig proteins are present in mammals: Insig-1 and Insig-2, which play a similar role in SREBP processing [10]. An earlier investigation indicated that the suppression of Insig2 during refeeding was the basic pathway of lipogenesis triggered by insulin. However, the remaining Insig-1 protein may be insufficient to block the processing and activation of SREBP [11, 12]. It is known that Insig2 has been informed to be connected to a variety of biological mechanisms, such as glucose and lipid metabolism [13–15] and immune response [16]. Since hepatic IR injury shows significant immune and metabolism turbulences in hepatocytes [17], it is necessary to clarify the regulatory function of Insig2 related to liver IR injury.

In the current investigation, we discovered that primary hepatocytes challenged with a hypoxia/reoxygenation (H/R) insult and liver exposed to IR surgery both had significantly lower levels of Insig2 expression. Furthermore, in order to evaluate the effect of Insig2 on hepatic IR injury in conjunction with the underlying mechanisms, we employed mice with global Insig2 knock-out (KO) and mice with hepatocyte-specific Insig2 overexpression. Additionally, our research revealed that Insig2 functioned as a protecting modulator against liver IR injury, as evidenced by both in vitro and in vivo studies. Furthermore, the complex molecular mechanisms underlying the protective effects of Insig2 against hepatic injury were obviously demonstrated——Insig2 interacts with the activation of the downstream pentose phosphate pathway (PPP). The supplementary PPP substrate could serve as an effective method for attenuating hepatic IR injury.

## Materials and methods

### Human liver tissues

Liver specimens were acquired from individuals experiencing orthotopic LT at ShuLan (Hangzhou) Hospital. Liver tissues were acquired from individuals' left lobes at two different time points: during back-table preparation (before transplantation) and approximately two hours after portal reperfusion (before abdominal closure). Moreover, all patients provided their written informed consent. The First Affiliated Hospital Ethics Committee at Zhejiang University School of Medicine in Hangzhou, China, gave its approval for this research, which complied with the 1975 Helsinki Declaration's ethical principles.

### Animals

The experiment involved the maintenance of male mice in a controlled environment that was free from pathogens and had a regulated temperature of 23 ± 2 °C. Additionally, the mice were subjected to a 12-h light/dark cycle. The experimental subjects were mice aged between 8 to 10 weeks and their average weight was measured to be 25 ± 2 g. The provision of nutrition in the form of food and water was available. The Zhejiang University animal care committee gave its approval to all animal procedures that were performed in accordance with the national academy of sciences protocol for the care and use of laboratory animals, which was made public by the national institutes of health (publication No. 85-23, revised 1985). Insig2 KO mice were provided by the laboratory of Di Wang, Zhejiang University School of Medicine. The identification primer sequences of Insig2-KO mice were F: 5′-GTGGGCTCTATGGCTTCTGA-3′ and R: 5′-AGGGTCCTTACCTGCAAACC-3′. Wild type mouse identification primer sequences were F: 5′-TGCATTGACAGGCATCTAGG-3′ and R: 5′-AGGGTCCTTACCTGCAAACC-3′. Moreover, genomic DNA was obtained from the newborn mice's toe tissue for examination by polymerase chain reaction (PCR). Additional file 1: Fig. S1 shows the result of genotyping.

### Mouse liver IR injury model

The study employed a mouse model that was subjected to partial (70%) warm IR injury. Moreover, the mice were anesthetized using Pentobarbital sodium at a dose of 50 mg/kg prior to performing a midline laparotomy. Consequently, the left lateral/median lobes of the liver were subjected to vascular occlusion by means of microvascular clips applied to the portal vein, the hepatic artery, and the bile duct above the point of bifurcation to the right lateral lobe. Following a period of 90 min of ischemia and 6 h of reperfusion, the subjects were euthanized. The sham control group of mice underwent an identical treatment, with the exception that their vasculature was not clamped. Following the trial, samples of serum and liver tissue were collected for subsequent analysis.

### Liver biochemical measurement

Utilizing Chemray 800 (Shenzhen, China) and the manufacturer's instructions, serum concentrations of the enzymes aspartate aminotransferase (AST) and alanine aminotransferase (ALT) were detected to evaluate mouse liver function. Employing commercial enzyme-linked immunosorbent assay (ELISA) kits (IL-6 Mouse Uncoated ELISA Kit, 88-7064-88; IL-1 beta Mouse Uncoated ELISA Kit, 88-7013-88; and TNF alpha Mouse Uncoated ELISA Kit, 88-7324-22 from Invitrogen) in accordance with the manufacturer's protocol, the inflammatory state was determined by quantifying serum cytokines.

### Histological and immunohistochemical staining

Hepatic tissue specimens were fixed in 10% formalin, dried, immersed in paraffin, and divided (5 μm thick) to determine the degree of liver necrosis. Consequently, hematoxylin and eosin (H&E) were employed to stain the sections. Immunohistochemistry (IHC) was utilized to assess Insig2 (24766-1-AP; Proteintech) and Casepase3 (19677-1-AP; Proteintech) expression. Two expert pathologists assigned IHC staining intensity grades of 0 (no staining), 1 (weakly positive), 2 (weak-moderate positive), 3 (moderately positive), 4 (moderate-strong positive), and 5 (strongly positive). Fluorescence microscopy (OLYMPUS; IX83) was utilized to take pictures.

### Immunofluorescence and terminal deoxynucleotidyl transferase-mediated deoxyguanosine triphosphate nick-end labeling (TUNEL) staining

The embedded liver segments in paraffin were also employed for immunofluorescence and TUNEL staining. In this study, primary antibodies targeting mouse MPO (22225-1-AP; Proteintech) and F4/80 (28463-1-AP; Proteintech) were employed. Furthermore, a secondary antibody, specifically goat anti-rabbit/mouse IgG-HRP (HKI0005; Haoke), was utilized. The TUNEL technique (HKI0008; Haoke) was utilized following the manufacturer’s instructions to identify the apoptosis in liver segments immersed in paraffin.

### Determination of glucose-6-phosphate dehydrogenase (G6PD) activity and related redox couples

Commercial kits (Beyotime, China) were employed following the manufacturer's directions to measure various parameters in hepatic tissues and hepatocytes, including G6PD activity, the reduced nicotinamide adenine dinucleotide phosphate/nicotinamide adenine dinucleotide phosphate (NADPH/NADP^+^), total Superoxide Dismutase (SOD) activity, glutathione/glutathione disulfide (GSH/GSSG), Malondialdehyde (MDA) levels and intracellular ROS production. The manufacturer's instructions were followed for all assays.

### Glucose and lactate concentration measurement

The concentrations of glucose, glucose-6-phosphate (G6P), and lactate in hepatic tissues were identified using an assay kit of glucose (Beyotime, China) and a lactate assay kit (Jiancheng Bio, Nanjing, China) following the protocol.

### Quantitative real-time PCR

Total mRNA was extracted from liver tissue and cultured cells using the Total RNA Isolation Kit (Yeason, China) according to the manufacturer’s directions and measured using a Nanodrop (ThermoFisher). Total RNA was used to synthesize cDNA with a SuperMix for qPCR Kit (Yeason). Consequently, quantitative real-time PCR was conducted with qPCR SYBR Green Master Mix (Yeason, China). The mRNA expression levels were standardized against β-actin expression. Additional file 1: Table S1 illustrates the primer sequences (Tsingke, Wuhan, China) of the target genes for real-time PCR.

### Western Blot analysis

In mouse hepatic tissue specimens and cells, western blot analysis was employed to identify protein expression levels. Total protein was isolated in radioimmunoprecipitation assay lysis buffer (Fdbio science), supplemented with protease inhibitor, protein phosphorylase inhibitors and PMSF (Fdbio science). The protein concentration was measured with a BCA Protein Assay Kit (Fdbio science). Next, after mixing with 5 × SDS loading buffer, protein supernatants were denatured at 95 °C for 10 min. Protein samples were separated by 10% SDS-PAGE, transferred to polyvinylidene fluoride membranes (IPVH00010, Millipore), blocked with 5% skim milk for 60 min, and incubated with primary antibodies at 4 °C overnight. Finally, membranes were incubated with the corresponding secondary antibodies. Images were obtained using Fluorescence Chemiluminescence Image Analyzer (ProteinSimple). β-actin served as an internal control. Additional file 1: Table S2 illustrates all antibodies used for western blot analysis.

### Cell culture, isolation of primary hepatocytes and hepatocyte hypoxia/reoxygenation (H/R) or hypoxia model

The primary hepatocytes and murine hepatocyte cell line AML12 (CRL-2254™) were used in this experiment. The two-step collagenase perfusion method was utilized to separate the primary hepatocytes of liver [18], and cells with > 80% viability were employed for more trials. Before cells were changed to sugar-free, serum-free Dulbecco’s modified Eagle’s medium (DMEM) for H/R experiments, they were cultivated in complete DMEM overnight. The establishment of cell environments (5% CO_2_, 1% O_2_ and 94% N_2_) was carried out using a modular incubator chamber (Astec APM-30D). To mimic liver IR in vivo, the cells underwent 6 h of hypoxia at the indicated time point before being restored to full medium and normal air conditions (95% air, 5% CO_2_) for 6 h.

The AML12 cells were cultured in DMEM/F-12 supplemented with 10% fetal bovine serum (FBS), ITS Liquid Media Supplement (PB180430, Procell), 40 ng/mL dexamethasone, and were maintained in a humidified incubator at 37 °C under 5% CO_2_ conditions. The hypoxic environment was simulated in vitro by using the hypoxia-inducing chemical cobalt chloride (CoCl_2_). Cells were cultured until they attained 60–70% confluence, and then cultured in FBS-free DMEM/F-12 with CoCl_2_ for 24 h. For further investigations, the above cells and related culture media were collected.

### Cell viability assay

Cell viability was detected using the CCK-8 kit (Fdbio). The cells were plated in 96-well plates at a 5000 cells/well density and incubated for 24 h. They were then subjected to hypoxia treatment (CoCl_2_); next, 10 μL of CCK8 solution was added to each well, followed by incubation for 1–6 h at 37 °C. Finally, the absorbance values of samples were measured at a wavelength of 450 nm using microplate reader (BioRad).

### Adeno-associated-virus 8 (AAV8) vectors construction and injection

AAV8 vectors for hepatocyte-specific Insig2 overexpression and knockdown were constructed by the Vigene Biosciences Co., Ltd (Shandong, China). In order to allow the selective expression of Insig2 in hepatocytes, the full-length Insig2 gene was cloned into an AAV8 vector with the thyroxine-binding globulin (TBG) promoter. The utilization of AAV8 vectors that express a triple short-hairpin RNA (shRNA) based on miR30 and were regulated by the TBG promoter was implemented for the purpose of Insig2 knockdown. The control group was subjected to the utilization of an AAV8-TBG vector, which contained a null cassette. By means of the tail vein, mice were administered with a virus containing 2 × 10^11^ AAV8 vector genomes in a volume of 100 μL.

### Small interference RNA (siRNA) transfection

AML12 cells were seeded in six-well plates at 2 × 10^5^ cells/well. After reaching approximately 70% confluence, the cells were transfected with the indicated siRNA (Tsingke, China) using Polyplus transfection reagent (jetPRIME) for 48 h. The sequences of siRNA duplexes were F: 5′-GUGCUAAAGUAGACUUCGATT-3′ and R: 5′-UCGAAGUCUACUUUAGCACTT-3′.

### Transcriptomics, proteomics and metabolomics analysis

Hepatic biopsy specimens were obtained from the Insig2-overexpression mice and control WT mice liver tissues subjected to IR injury. Consequently, sample preparation and extraction for transcriptomics, proteomics, and metabolomics sequencing was performed by Metware Biotechnology Co., Ltd. (Wuhan, China). Moreover, on the Illumina sequencing platform, the cDNA libraries were sequenced for transcriptomics. Furthermore, genes greater than 2.0-fold change and P value < 0.05 between two groups were regarded as differentially expressed genes (DEGs). The Kyoto Encyclopedia of Genes and Genomes (KEGG), GeneOntology (GO), and Metascape online databases were utilized for pathway enrichment analysis of differential genes. For proteomic analysis, the Swissprot.Mouse.20200826.fasta database was used for protein identification. In particular, differentially expressed proteins (DEPs) were those whose expression levels were greater than 1.2-fold change and P value < 0.05. The enrichment study of differential proteins was performed utilizing the internet databases including, KEGG, GO, and Metascape. Additionally, the Metware database was utilized to identify metabolites for metabolomics study. Orthogonal projections to latent structure-discriminant analysis (OPLS-DA) score plots were employed to show the data's intrinsic differences. Differentially abundant metabolites (DEMs) were significantly distinguished using variable importance in projection (VIP) > 1 and P values < 0.05. Metabolites with significant differences between the two groups were used for KEGG enrichment analysis using MetaboAnalyst v.5.0.

### Seahorse metabolic analysis

The Extracellular acidification rate (ECAR) was determined using Seahorse XF Glycolysis Stress Test Kit (103,344–100, Agilent Technologies). AML12 cells were plated in XF96-well culture plates at a density of 5 × 10^3^—1 × 10^4^ cells/well and incubated at 37 °C overnight. Subsequently, the cells were washed with assay medium (103,575–100, XF DMEM Medium) and then placed in a CO_2_-free incubator at 37 °C for 1 h before the measurement. To measure ECAR, three injections of specific substances (glucose at 10 mM, oligomycin at 1 μM and 2-DG at 50 mM) were administrated at the designated timepoints. The data were analyzed using Seahorse Wave software (Seahorse Bioscience, Agilent Technologies).

### Nanoparticle delivery system

Herein, we demonstrate a mature nanoparticle for effective delivery of D-glucose 6-phosphate disodium salt, which is a polyplex of G6P, esterase-responsive polymer (ERP) and DC-Chol/DOPC lipids [19, 20]. In summary, the G6P disodium salt was prepared by dissolving it in HEPES at a 5 mg/mL concentration to obtain a stock solution. The ERP was dissolved in a DMSO buffer at 50 mg/mL concentration and used as a stock solution. The pH 7.4 HEPES buffer solution (10 mM) was diluted to the necessary concentrations using the stock solutions, based on the mass ratios. At room temperature, equal volumes of ERP and G6P were incubated for 30 min after mixing and vortexed for 10 s. Consequently, at 25 °C, Zetasizer Nano-ZS (Malvern Instruments) was employed to detect the sizes and zeta potentials of polyplexes in the HEPES buffer. As described above, ERP/G6P polyplexes were prepared at an optimized mass ratio of 0.1 (Additional file 1: Fig. S2). The fabrication of ERP/G6P polyplexes coated with lipids (DC-Chol/DOPC) conjugated to DSPC-PEG-Gal was synthesized according to the previous report. A thin lipid film was obtained by dissolving neutral-charged helper lipids DOPC and cationic lipids DC-Chol (1:1) in chloroform, then evaporating the chloroform. The HEPES buffer was supplemented and stirred for 8 h at room temperature, followed by 10 min of sonication. Subsequently, the ERP/G6P polyplex solution was added dropwise to the lipid solution and stirred for a further 2 h at room temperature. Finally, DSPE-PEG-Gal was added to the above mixture and stirred for 15 min to obtain PEG-Lipids/ERP/G6P at 50 °C. At 25 °C, a Zetasizer Nano-ZS (Malvern Instruments) was employed to detect the sizes and zeta potentials of PEG-Lipids/ERP/G6P (Additional file 1: Fig. S3) to optimize the final concentration (DOPC/G6P [μM/μg] = 0.01; 5%PEG). The biodistribution of PEG-Lipids/ERP/G6P was investigated by tracking the DiD-loaded PEG-Lipids/ERP/G6P after intravenous (IV) injection for different times. The main organs (heart, liver, spleen, lung and kidney) were dissected at 2, 6, 12, 24, 48 and 72 h post-injection and imaged with the DiD fluorescence intensity. The D-glucose 6-phosphate disodium salt was purchased from Selleck (CAS: 3671-99-6). Utilizing the tail vein, mice were administered with G6P or G6P nanoparticles in a volume of 200 μL for 2 consecutive days.

### Statistical analysis

All continuous data are presented as the mean ± SD, and categorical data are presented as frequency or numbers. The unpaired two-tailed Student *t*-test was employed to analyze normally distributed data between two groups, while one-way analysis of variance (ANOVA) was employed to compare data between several groups, and appropriate post hoc test was used to test difference between groups. The data that doesn’t conform to a normal distribution will be analyzed using the Wilcoxon test. Statistics were deemed significant at P < 0.05. All statistical analyses were performed using the SPSS program (version 21.0). The P values are as follows: *P < 0.05; **P < 0.01.

## Results

### Insig2 expression is significantly down-regulated during hepatic IR injury and negatively correlates with IR-induced liver injury in patients

**Fig. 1:**
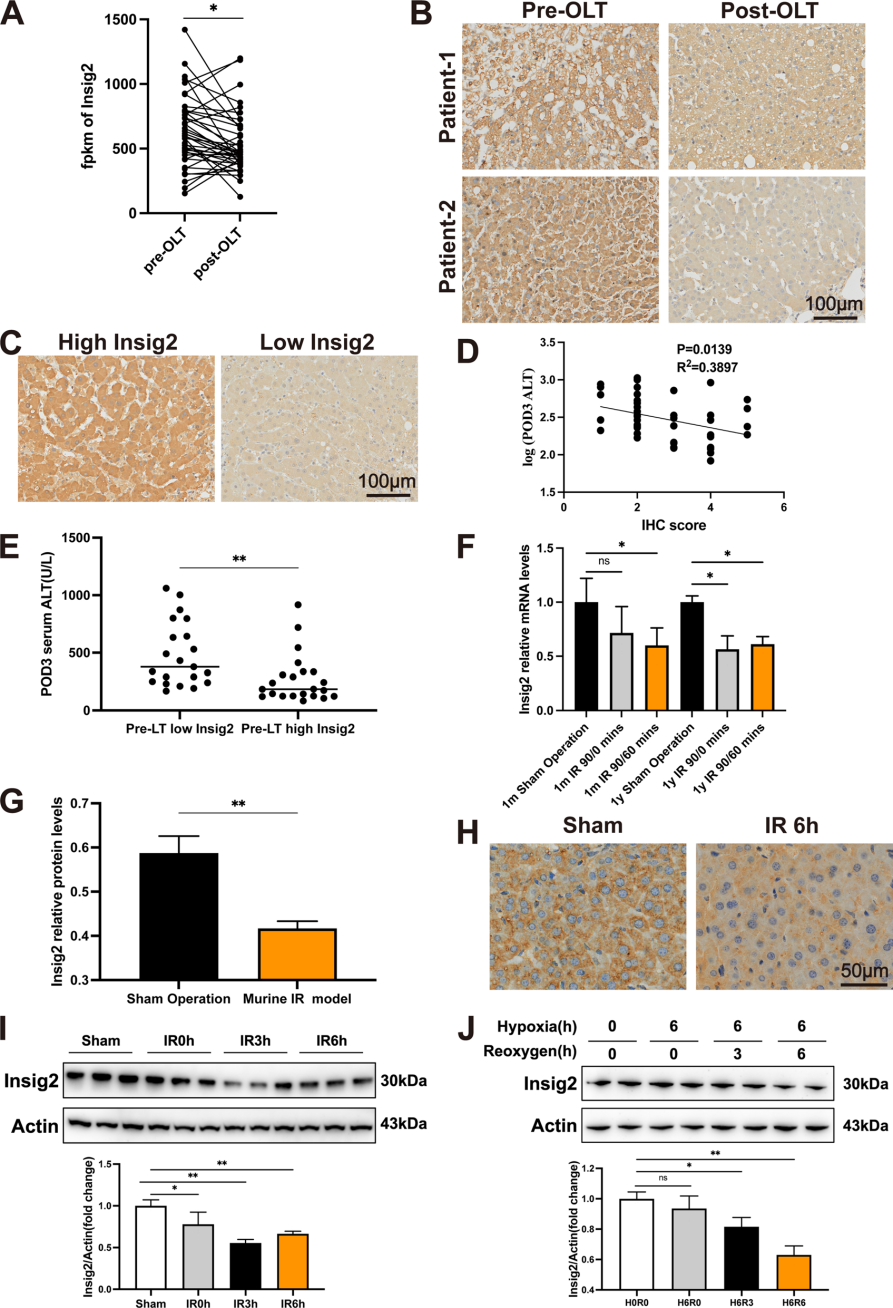
Insig2 expression is decreased in hepatic IR injury and negatively correlates with IR-induced liver injury in patients. **A** Insig2 mRNA levels in 40 donor livers of persons subjected to OLT from GSE151648 database. **B** Representative IHC staining of Insig2 in liver sections of persons undergone LT. **C** 43 human OLTs were divided into low (n = 21) and high (n = 22) Insig2 expression group of pre-OLT livers using IHC scores (IHC score < 3 as the threshold). **D** Pearson’s correlation coefficient between the pre-OLT livers’ IHC scores and serum ALT (sALT) at POD3 (R^2^ = 0.3897, P = 0.0139). **E** sALT values in both low-Insig2 and high-Insig2 groups in OLT recipients at POD3. **F** Insig2 mRNA levels in livers of WT mice (1 m or 1y old) subjected to 90 min of ischemia and subsequent reperfusion for 0, 60 min from GSE10657 database (n = 3/time point). **G** Insig2 protein expression in livers of WT mice subjected to 1.5 h of ischemia and subsequent reperfusion for 6 h (n = 3/group) based on proteomics-sequencing. **H** Representative IHC staining of Insig2 in livers of mice subjected to sham treatment or hepatic IR surgery. **I** Western blot analysis of Insig2 protein expression in livers from WT mice subjected to sham treatment or ischemia for 1.5 h, followed by reperfusion for 0, 3 and 6 h (n = 3/group). **J** Western blot analysis of Insig2 protein expression in primary hepatocytes of WT mice subjected to 6 h of hypoxia and subsequent reoxygenation for 0, 3 or 6 h (n = 2/group). All data are shown as means ± SDs. ns indicates no significance between the two indicated groups; *p < 0.05; **p < 0.01

To explore the Insig2 function in the pathogenesis of hepatic IR, we first tested Insig2 expression levels in liver specimens from 40 patients undergoing orthotopic liver transplantation (OLT) from GSE151648. The mRNA level of Insig2 in the reperfusion specimens was significantly decreased in contrast to the baseline levels (Fig. 1A). Additionally, the IHC staining test revealed that hepatocytes were the primary site of this reduction in Insig2 (Fig. 1B). In addition, we divided the patients into low-Insig2 and high-Insig2 groups using IHC scores based on preoperative donor livers (Fig. 1C). Interestingly, preoperative Insig2 levels were negatively correlated with serum ALT (sALT) values at POD3 (Fig. 1D: R^2^ = 0.3897, p < 0.05). Patients with high-Insig2 levels exhibited lower levels of sALT in POD3 compared to the low-Insig2 group (Fig. 1E). In livers of hepatic IR injury model mice, we also observed constant decreases in Insig2 mRNA according to the GSE10657 database (Fig. 1F) and protein levels according to IHC, proteomics sequencing, and Western blot results (Fig. 1G–I), in accordance with those of clinical samples. In an in vitro H/R model utilizing primary hepatocytes obtained from wild-type (WT) mice, the Insig2 downregulation in hepatocytes was further verified (Fig. 1J).

### Insig2 deficiency aggravates hepatic IR injury by promoting inflammation and apoptosis

**Fig. 2:**
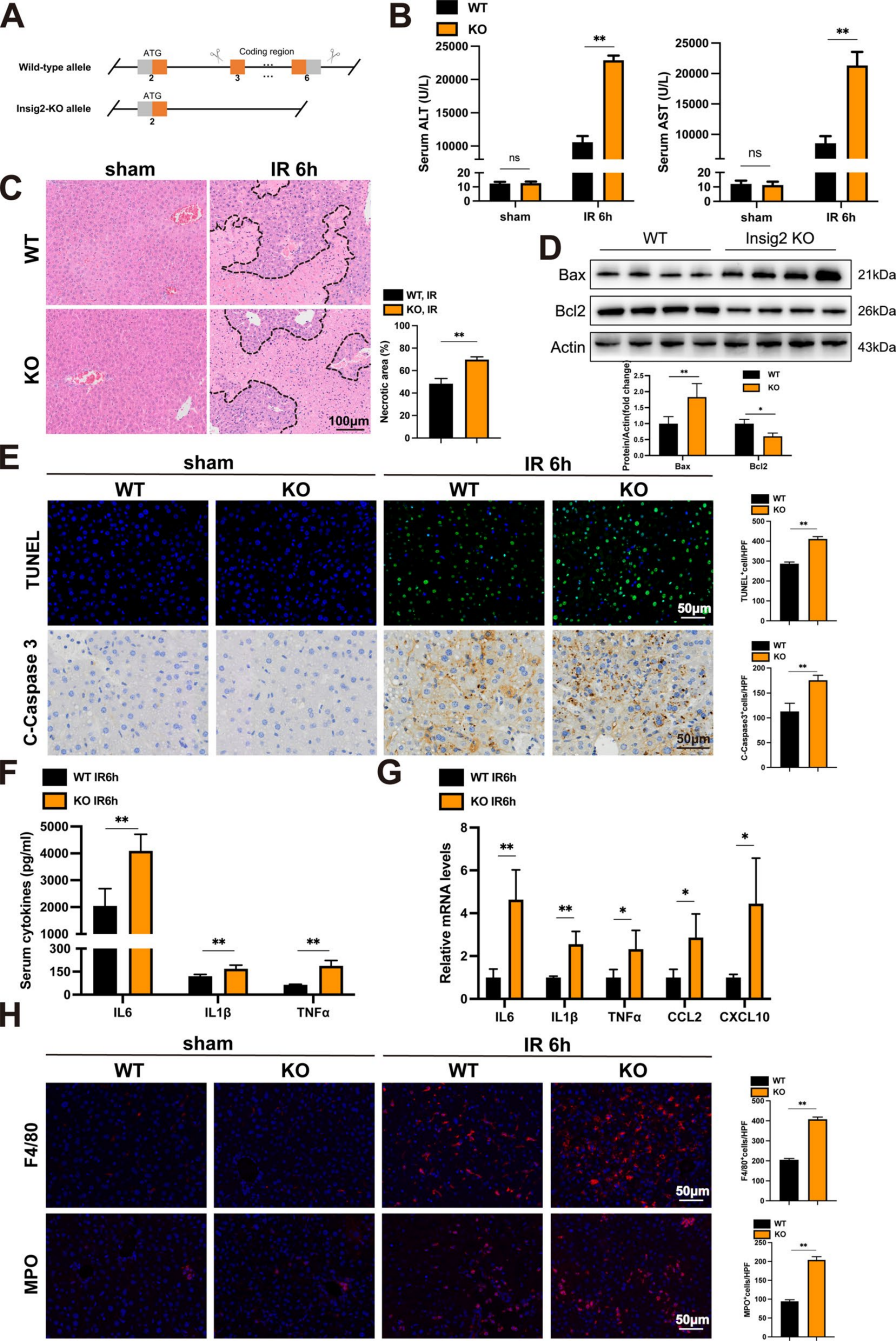
Insig2 deficiency exacerbates hepatic IR injury. **A** Diagram of the Insig2-KO strategy. **B** sALT and sAST levels in WT and Insig2-KO mice in the sham group (n = 3/group) and at 6 h after hepatic IR (n = 6/group). **C** Representative HE staining and statistics showing necrotic areas of liver tissue from WT and Insig2-KO mice at 6 h after hepatic IR (n = 3/group). **D** Western blot analysis of Bax and Bcl2 expression in livers from WT and Insig2-KO mice at 6 h after IR (n = 4/group). **E** TUNEL staining and statistics in ischemic liver sections from mice in the indicated groups (n = 3/group). IHC staining of cleaved-caspase-3 expression and statistics in ischemic liver sections from mice in the indicated groups (n = 3/group). **F** The serum levels of inflammatory cytokines (IL-6, IL-1β, TNF-α) in WT and Insig2-KO mice at 6 h after hepatic IR (n = 5/group). **G** Real-time quantitative PCR (RT-PCR) analysis of the mRNA levels of IL-6, IL-1β, TNF-α, Ccl2 and Cxcl10 in WT and Insig2-KO mice at 6 h after hepatic IR (n = 4/group). **H** Representative MPO and F4/80 immunofluorescent staining (red) in liver sections of WT and Insig2-KO mice at sham or IR surgery (n = 3/group). All data are shown as means ± SDs. ns indicates no significance between the two indicated groups; *p < 0.05; **p < 0.01

To investigate the Insig2 role in IR-induced liver injury, we utilized Insig2-KO mice (Fig. 2A) and subjected them to IR surgery alongside WT mice. At 6 h post-IR, serum levels of ALT and AST were significantly higher in Insig2-KO mice than in WT mice (Fig. 2B). The histological assessment revealed that Insig2-KO mice had significantly greater necrotic regions than WT mice (Fig. 2C). At 6 h after IR, our western blot results verified the down-regulation of the anti-apoptotic factor B-cell leukemia/lymphoma (Bcl-2), and the up-regulation of the proapoptotic factor Bcl2-associated X protein (Bax) in the livers of Insig2-KO mice versus WT mice (Fig. 2D). Following IR injury, we identified apoptosis of hepatocytes employing TUNEL and C-Casepase3 IHC staining in Insig2-KO and WT mice. Figure 2E demonstrated that Insig2-KO significantly aggravated cell apoptosis. During the reperfusion phase, the main manifestations and pathogenic factors are the release of inflammatory cytokines. Figure 2F demonstrated that IL-6, IL-1β, and TNF-α serum levels were significantly elevated in the Insig2 KO group in contrast to the control group, as determined by ELISA for testing these main cytokines expression. The Insig2-KO group also had significantly higher mRNA levels of the proinflammatory cytokines and chemokines IL6, IL1β, TNF-α, C–C motif chemokine ligand 2 (Ccl2), and CXC motif chemokine ligand 10 (Cxcl10) in hepatic tissues (Fig. 2G). Additionally, the IF test revealed that Insig2-KO mice had significantly greater infiltration levels of MPO-positive and F4/80-positive inflammatory cells in their livers than WT mice (Fig. 2H). Collectively, Insig2 deficiency aggravates hepatic IR-induced apoptosis and inflammation.

### Hepatocyte-specific Insig2 overexpression inhibits IR injury

**Fig. 3:**
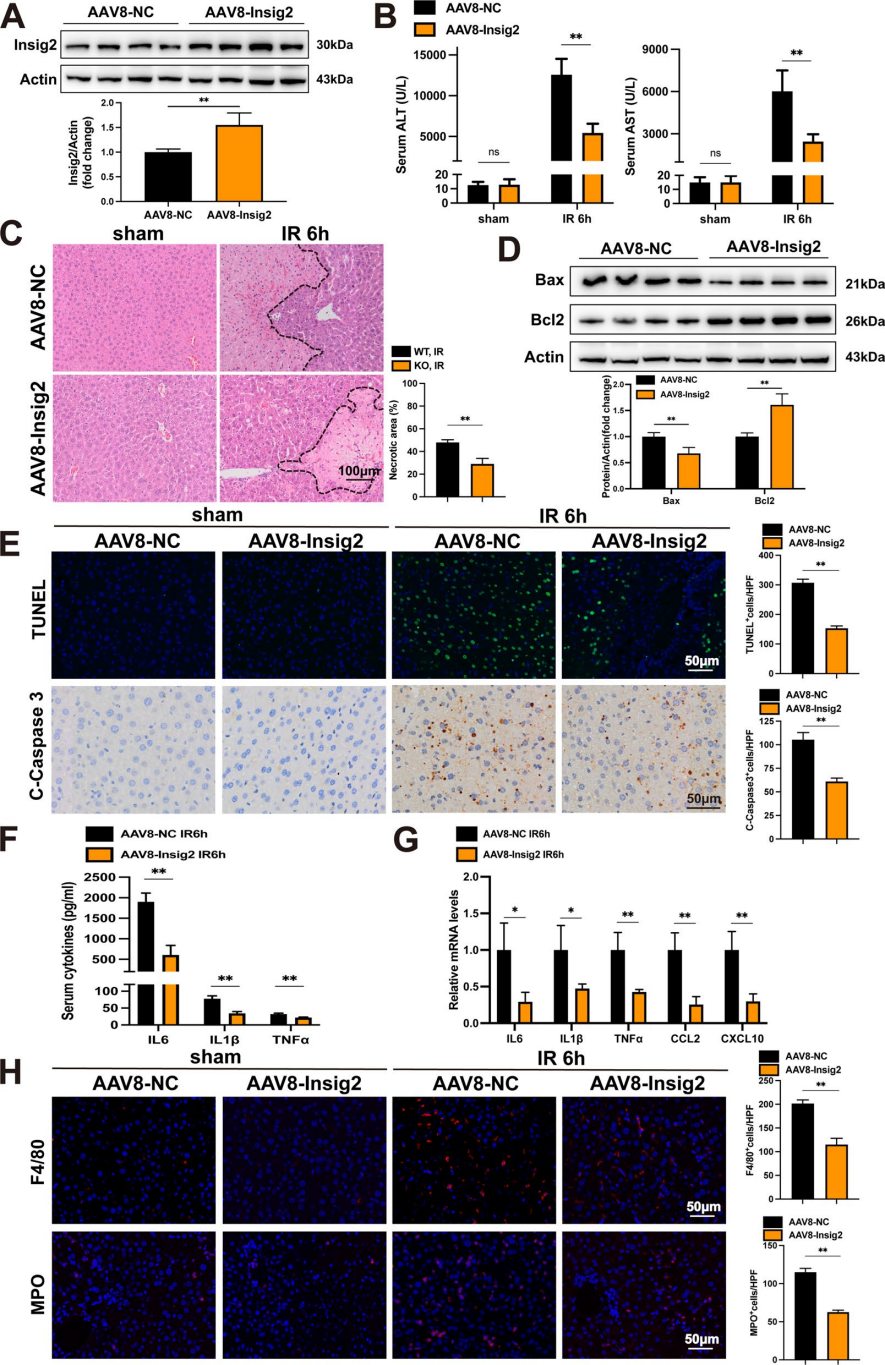
AAV8-mediated hepatic Insig2 overexpression ameliorates hepatic IR injury. **A** The AAV8 transfection efficiency was confirmed by western blot using liver tissue from mice. The WT mouse infected by AAV8 carrying Insig2 (AAV8-Insig2) or the control virus (AAV8-NC). **B** Levels of serum ALT and AST in AAV8-NC and AAV8-Insig2 mice under sham treatment (n = 3/group) and at 6 h after hepatic IR (n = 6/group). **C** Representative HE staining and quantification of necrotic areas of liver tissue from AAV8-NC and AAV8-Insig2 mice at 6 h after reperfusion or sham treatment (n = 3/group). **D** Western blot analysis of Bax and Bcl2 expression in livers from AAV8-NC and AAV8-Insig2 mice at 6 h after reperfusion (n = 4/group). **E** Representative TUNEL staining, IHC staining of cleaved-caspase-3 expression of liver tissues from AAV8-NC and AAV8-Insig2 mice at 6 h after reperfusion (n = 3/group). **F** The serum levels of inflammatory cytokines (IL-6, IL-1β, TNF-α) in AAV8-NC and AAV8-Insig2 mice at 6 h after reperfusion (n = 5/group). **G** RT-PCR analysis of the mRNA levels of IL-6, IL-1β, TNF-α, Ccl2 and Cxcl10 in AAV8-NC and AAV8-Insig2 mice at 6 h after reperfusion (n = 4/group). **H** Representative MPO and F4/80 IF staining (red) in liver sections of AAV8-NC and AAV8-Insig2 mice at sham or IR surgery (n = 3/group). All data are shown as means ± SDs. ns indicates no significance between the two indicated groups; *p < 0.05; **p < 0.01

The present investigation has demonstrated that the overexpression of Insig2 could potentially serve as a protective mechanism against hepatic IR injury. In order to achieve this objective, we employed AAV8-Insig2 injections administered through the tail vein to induce overexpression of Insig2 in the liver of mice. In parallel, AAV8-green fluorescent protein (GFP)-infected mice were employed as controls (Fig. 3A), and their reaction to an IR injury was subsequently assessed. Moreover, our findings demonstrated that serum ALT and AST levels in Insig2-overexpression (Insig2-OE) mice were significantly lower than those of control mice 6 h after IR (Fig. 3B). Furthermore, it was observed that Insig2-OE mice exhibited a significant decrease in liver necrotic regions in comparison to the control group (Fig. 3C). The results indicated that the overexpression of Insig2 led to a significant reduction in cell death and related protein expression, as evidenced by the outcomes of TUNEL and C-Casepase3 staining, as well as western blot analysis (Fig. 3D–E). In addition, the Insig2-OE mice exhibited a significant decrease in serum cytokine and cytokine mRNA levels in liver tissues (Fig. 3F–G). Hepatic infiltration of inflammatory cells (MPO^+^, F4/80^+^) was significantly lowered in Insig2-OE mice (Fig. 3H). These outcomes revealed that hepatic Insig2 overexpression alleviated cell death, inflammation, and hepatic damage during IR injury.

### Insig2 protected hepatocytes from H/R or hypoxia-induced cell inflammation and apoptosis

**Fig. 4:**
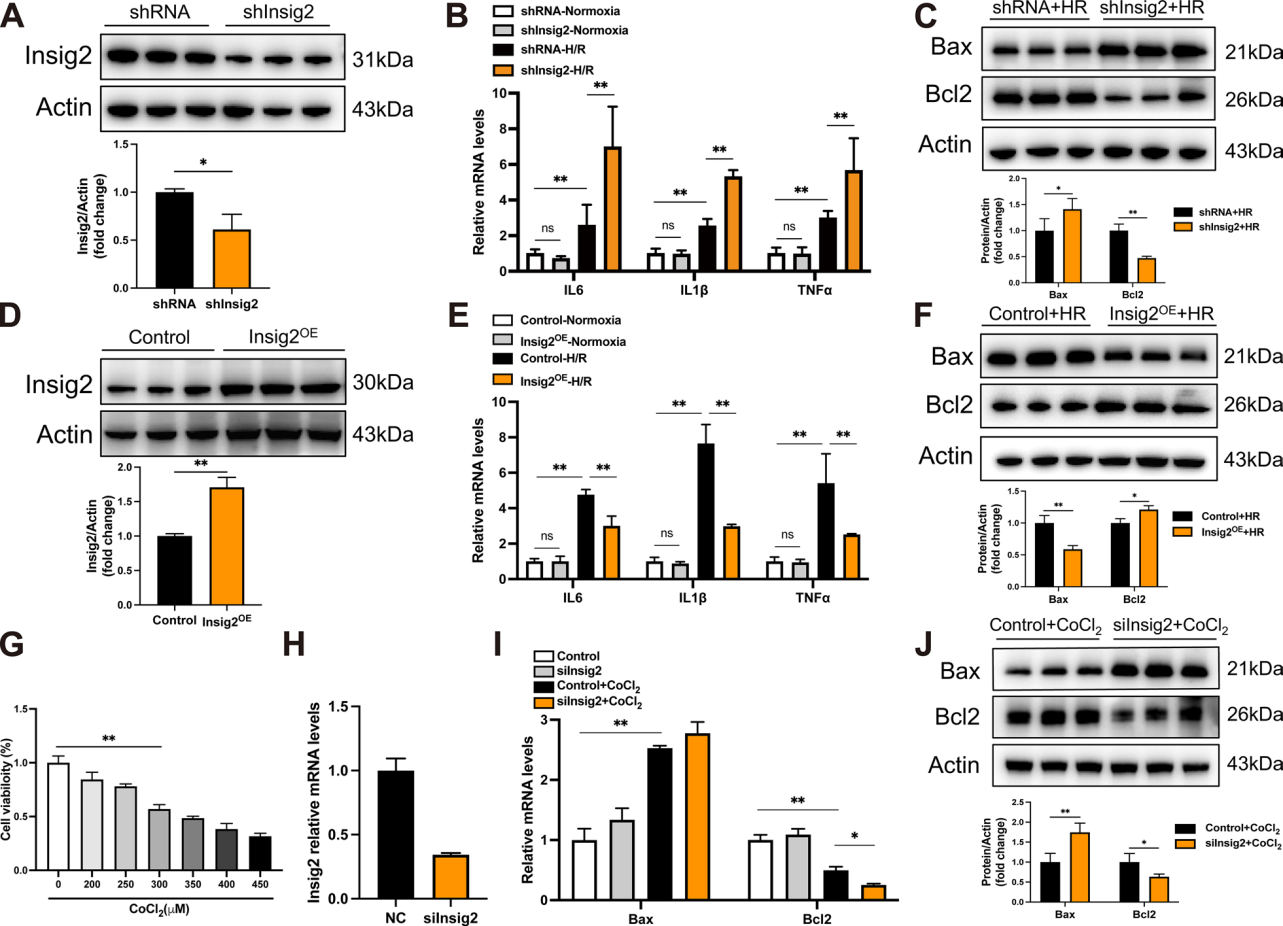
Insig2 alleviates inflammation and apoptosis during hepatic H/R. **A** Insig2 protein expression in primary hepatocytes extracted from mice infected with shRNA or shInsig2 AAV8. **B** mRNA levels of proinflammatory factors (IL-6, IL-1β, TNF-α) in hepatocytes from the indicated groups after H/R challenge (n = 3/group). **C** Expression of apoptosis signaling pathway proteins in hepatocytes from the indicated groups after H/R challenge (n = 3/group). **D** Insig2 protein expression in primary hepatocytes extracted from mice infected with Control or Insig2 overexpressing AAV8. **E** mRNA levels of proinflammatory factors (IL-6, IL-1β, TNF-α) in hepatocytes from the indicated groups after H/R challenge (n = 3/group). **F** Expression of apoptosis signaling pathway proteins in hepatocytes from the indicated groups after H/R challenge (n = 3/group). **G** The AML12 cells were subjected to the CCK-8 assay to determine the appropriate concentration of CoCl_2_. **H** Establishment of Insig2 knockdown AML 12 cell lines (n=3/group). **I**–**J** The mRNA and protein levels of Bax, Bcl2 in AML12 cells from the indicated groups (n=3/group). All data are shown as means ± SDs. ns indicates no significance between the two indicated groups; *p < 0.05; **p < 0.01

The present study involved an evaluation of the role of Insig2 in responding to the H/R challenge. Primary hepatocytes were obtained from mice that had been injected with AAV8 for knockdown or overexpression of Insig2 at a consistent age. To identify inflammation and cell-apoptosis-related signaling, hepatocytes were treated with hypoxia for 6 h, followed by 6 h of reoxygenation. Following the H/R challenge, a comparison of the result revealed that the knockdown of Insig2 had a significantly detrimental effect on cellular inflammation (Fig. 4B). Additionally, Bcl2 was significantly diminished by Insig2 knockdown, but Bax was significantly upregulated (Fig. 4C). On the other hand, Insig2 overexpression significantly reduced the H/R-induced increase in inflammation and apoptosis as a result of H/R treatment (Fig. 4D–F). To establish a CoCl_2_-induced hypoxia model, we exposed AML12 cells to varying concentrations of CoCl_2_ for 24 h, followed by a CCK-8 assay (Fig. 4G) to assess cell viability. CoCl_2_ treatment exerted a significant impact on cell viability, leading to a substantial reduction. Notably, at a concentration of 300 μmol/L CoCl_2_, the cell survival rate was approximately 50%. Based on this observation, we selected this specific concentration for further experiments. Next, we generated an Insig2 knockdown cell line to investigate the role of Insig2 in hepatocyte apoptosis under hypoxic conditions (Fig. 4H). The results demonstrated that Insig2 knockdown increased the expression of Bax and reduced the expression of Bcl2, both at the mRNA and protein levels (Fig. 4I, J). Ultimately, these outcomes showed that Insig2 had the ability to protect the primary hepatocytes against inflammation and death caused by H/R or hypoxia.

### The combined analysis of transcriptomics, proteomics, and metabolomics reveals the key downstream mechanism

**Fig. 5:**
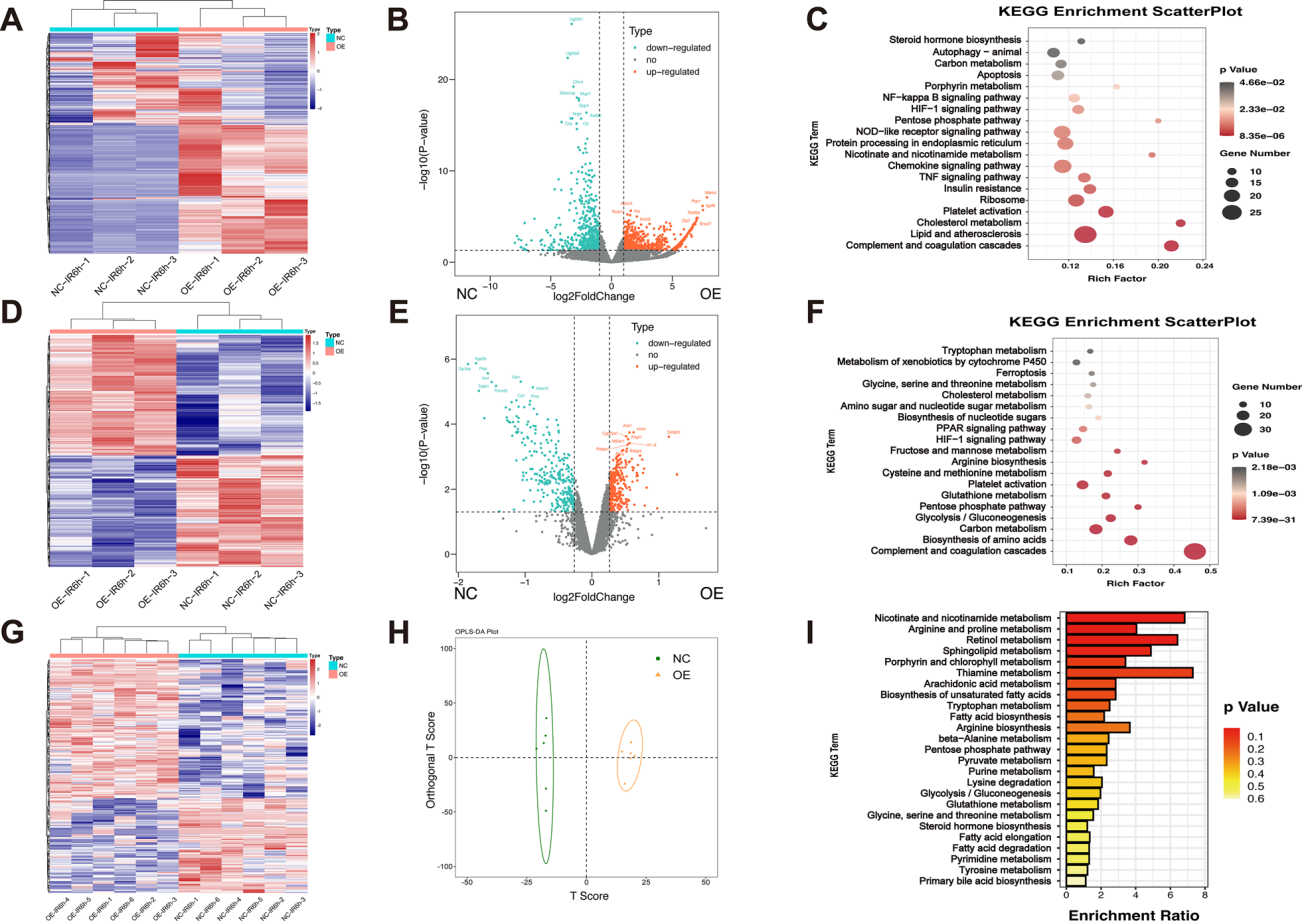
Transcriptome, proteome, and metabolome network analysis reveals disturbed pathways in Insig2 overexpression mice relative to those in WT controls subjected to IR injury. **A** Hierarchical clustering heat map showing the distribution profiles of RNA-seq (n = 3/group). **B** Volcano plots indicating DEGs (red, up-regulated genes; blue, down-regulated genes). **C** Major biological pathways contributing to Insig2 function based on KEGG enrichment analysis of RNA-seq. **D** Hierarchical clustering heat map showing the distribution profiles of proteomics (n = 3/group). **E** Volcano plots indicating DEPs (red, up-regulated proteins; blue, down-regulated proteins. **F** Major biological pathways contributing to Insig2 function based on KEGG enrichment analysis of proteomics. **G** Hierarchical clustering heat map showing all detected DEMs (n = 6/group). **H** OPLS-DA image showing global sample distribution profiles. **I** Pathway-based analysis of metabolic changes

To investigate the underlying pathways and pathogenesis that account for the Insig2’s protective role against hepatic IR injury, we conducted an integrated analysis of transcriptomics, proteomics, and metabolomics in liver tissues from Insig2-OE mice and controlled WT mice following IR injury. A hierarchical clustering dendrogram study revealed the RNA-sequencing distribution profiles (Fig. 5A). Volcano mapping showed 782 significantly upregulated DEGs (fold change > 2, p < 0.05) and 599 downregulated DEGs (Fig. 5B). The KEGG pathway enrichment analysis indicated that the insulin resistance, cholesterol metabolism pathway, and PPP were significantly enriched signaling mechanisms connected with the DEGs during IR (Fig. 5C). The GO pathway enrichment study indicated that the response to oxidative stress (OS) and lipid metabolism-associated mechanism and related genes were down-regulated by Insig2 overexpression (Additional file 1: Fig. S4).

A proteomic study was performed to verify the transcriptome profiling outcomes. Significant variations were exhibited by a hierarchical clustering heat map of all proteins detected in the two groups (Fig. 5D). A total of 318 DEPs (fold change > 1.2, p < 0.05) were significantly upregulated, and 294 proteins were downregulated in the Insig2-OE liver (Fig. 5E). The KEGG pathway enrichment analysis demonstrated that glucose and lipid metabolism pathways, such as PPP, were enriched and associated with the DEPs during IR (Fig. 5F). The GO enrichment analysis further confirmed that OS and lipid metabolism were among the main differentially expressed mechanisms, consistent with the transcriptome analysis (Additional file 1: Fig. S4).

Therefore, the metabolic activities were considerably varied between both groups. Metabolomics profiles could easily distinguish Insig2-OE liver samples using OPLS-DA models (Fig. 5G, H). There were 259 DEMs (105 lower and 154 higher, VIP > 1, p < 0.05) among the annotated metabolites in these samples. The abundances of many amino acids and derivatives, nucleotides and derivatives, and organic acid metabolites were significantly different. The KEGG enrichment analysis showed that glycolysis and PPP pathways were different between the two groups (Fig. 5I).

Combined analysis of DEGs, DEPs, and DEMs between Insig2-OE and controlled WT by KEGG showed that the activation of PPP was the only differential pathway. The clustering heat maps representing PPP-related genes and proteins revealed 6 DEGs and 9 DEPs that exhibited differential expression upon Insig2 overexpression, with the majority of them being upregulated. The PPP activation was evident through the decreased Gluconic acid and increased 2-Deoxyribose-5′-phosphate in metabolomics (Additional file 1: Fig. S5). All these findings served as additional evidence that Insig2 played a role in reinvigorate and promote the previously inhibited PPP biosynthetic pathways under the condition of IR injury.

### Insig2 mediates the activation of pentose phosphate pathway and inhibition of oxidative stress in hepatic IR injury

**Fig. 6:**
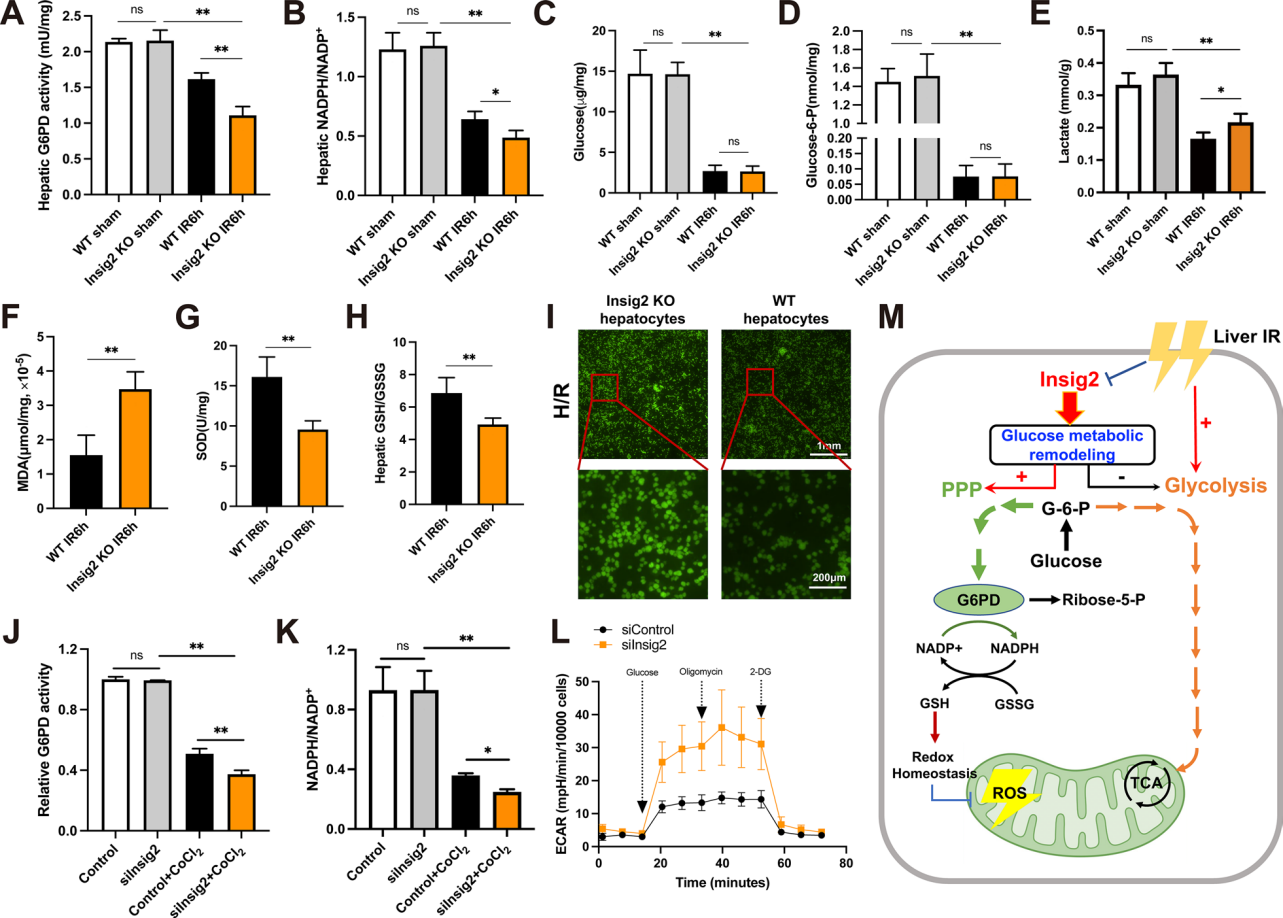
Insig2 maintained active pentose phosphate pathway (PPP) metabolism and redox homeostasis. **A**–**E** G6PD activity (n = 6/group), NADPH/NADP^+^ ratio (n = 5/group), glucose (n = 6/group) and G6P (n = 6/group) consumption, lactate production (n = 6/group) in liver tissues were detected at sham/IR group from Insig2 deficiency and WT mice. **F**–**H** The MDA level, SOD activity, GSH/GSSG ratio were detected at IR group from Insig2 deficiency and WT mice (n = 6/group). **I** The ROS fluorescence photographs of Insig2-KO and controlled WT primary hepatocytes subjected to H/R. **J**–**L** G6PD activity (n = 3/group), NADPH/NADP^+^ ratio (n = 3/group) and measurement of the ECAR (n = 3/group) in control or Insig2 knockdown AML12 cells upon CoCl_2_-induced hypoxia. **M** Schematic depicting the mechanisms by which Insig2 regulates the PPP and glycolysis in hepatocytes during hepatic I/R injury. All data are shown as means ± SDs. ns indicates no significance between the two indicated groups; *p < 0.05; **p < 0.01

The G6P produced in the cytosol can generate NADPH via the oxidative phase of PPP, and NADPH is a main component of the cellular antioxidant defense system. Following the beneficial effects of Insig2 on hepatic IR injury, we further tested whether the metabolites and enzymes in glucose metabolism were altered by Insig2 deficiency. To this end, the results showed that the activity of G6PD, the rate-limiting enzyme in the PPP, was significantly decreased in liver tissues with Insig2 deficiency (Fig. 6A). This change in intracellular redox balance was associated with a lower NADPH/NADP^+^ ratio (Fig. 6B). The consumption of glucose and G6P increased after IR was independent of Insig2, and the lactate content increased under the condition of Insig2 deficiency (Fig. 6C–E). Inhibition of PPP significantly increased OS damage and reduced antioxidant enzyme activities of hepatocytes. A product of lipid peroxidation (MDA), two endogenous free-radical scavengers (SOD, GSH) are common and important markers of OS. As expected, Insig2 deficiency elevated the MDA content and decreased the activity of SOD and the ratio of GSH (reduced)/GSSG (oxidized) compared to those of the WT group (Fig. 6F–H). In vitro experiments, primary hepatocytes with Insig2 deficiency showed a significant increase in the fluorescence intensity of ROS when subjected to H/R stimulation (Fig. 6I). Additionally, we explored whether Insig2 could affect hepatic glycolytic metabolism, as any factor impacting the PPP had the potential to alter glucose flux in glycolysis. The results revealed that AML12 cells with Insig2 knockdown exhibited a decline in G6PD activity and a reduction in the NADPH/NADP^+^ ratio upon exposure to CoCl_2_-induced hypoxia (Fig. 6J–K). Moreover, the measurement of ECAR levels indicated that Insig2 knockdown significantly increased glycolytic activity in AML12 cells subjected to CoCl_2_-induced hypoxia (Fig. 6L). Together, these outcomes suggested that Insig2 played a crucial role in glucose metabolic remodeling, promoting the glucose flux shifting more toward the PPP, which contributes to redox homeostasis (Fig. 6M).

**Fig. 7:**
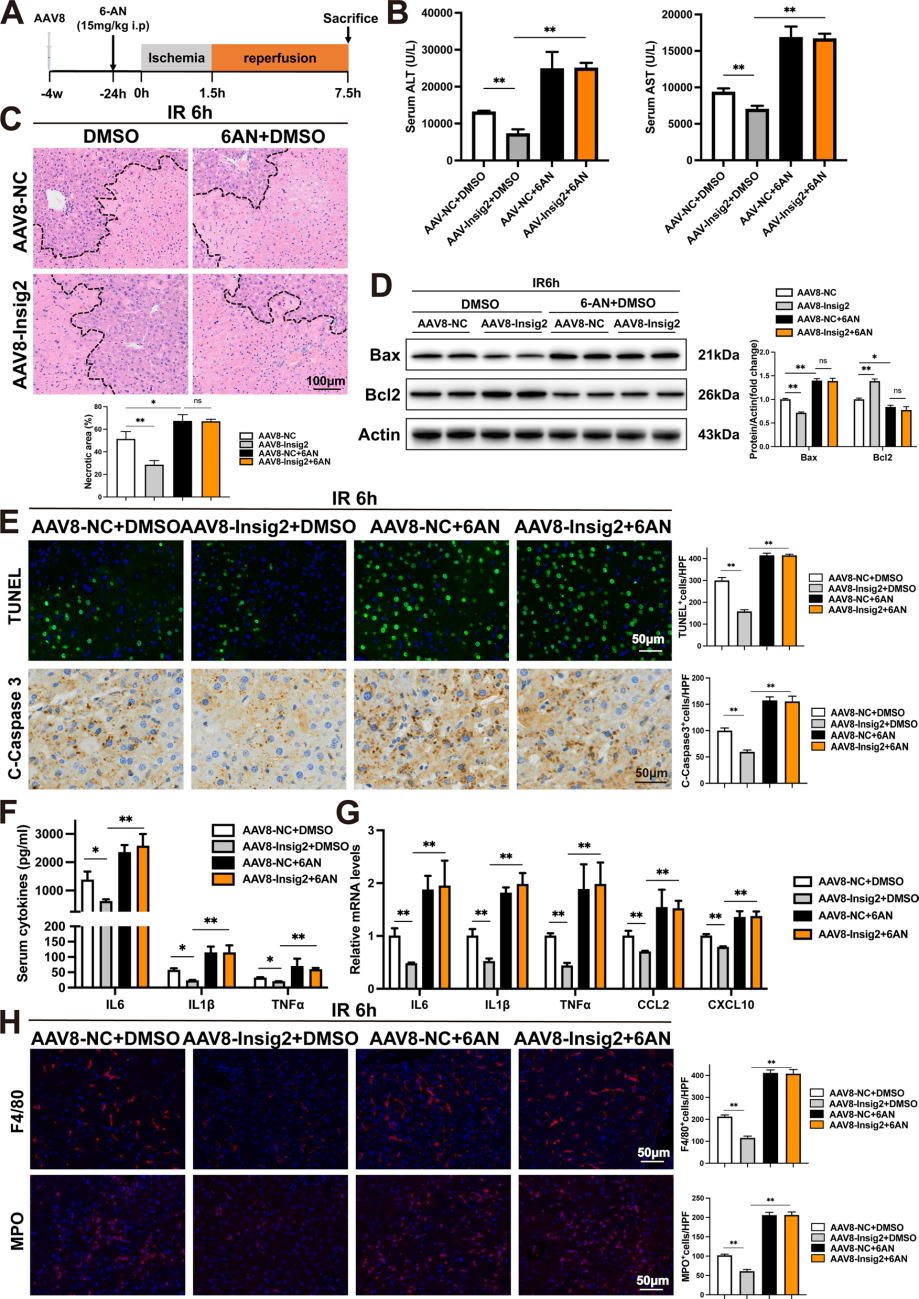
The PPP mediates the effect of Insig2 on hepatic IR injury. Mice injected with AAV8-NC and AAV8-Insig2 were treated with DMSO or 6-AN, followed by hepatic IR injury. **A**, **B** Levels of serum ALT and AST (n = 3/group). **C** Representative HE staining and quantification of necrotic areas (n = 3/group). **D** Expression of apoptosis signaling pathway proteins in liver tissues (n = 2/group). **E** Representative TUNEL staining, IHC staining of cleaved-caspase-3 expression of liver tissues (n = 3/group). **F**–**G** Serum and hepatic levels of proinflammatory cytokines/chemokine (n = 3/group). **H** Representative MPO and F4/80 IF staining in liver sections (n = 3/group). All data are shown as means ± SDs. ns indicates no significance between the two indicated groups; *p < 0.05; **p < 0.01

To further evaluate whether Insig2’s impact on hepatic IR injury and OS occurring was dependent on PPP, PPP activity was blocked in vivo using the particular G6PD suppressor 6-Aminonicotinamide (6-AN, Selleck, CAS: 329-89-5) as the previous study [21]. Consequently, we injected Insig2-OE and controlled WT mice with 6-AN or DMSO and constructed IR models in the four groups (Additional file 1: Figs. S6A-B). Importantly, 6AN largely abolished Insig2-OE-induced alleviation of hepatic damage at 6 h postreperfusion (Fig. 7A–C). Upon inhibiting the PPP, the downstream OS indicators (GSH/GSSG, SOD, MDA) were found to be activated, leading to a disturbance in the redox balance (Additional file 1: Figs. S6C–E). Inhibition of G6PD also altered Insig2-OE-triggered decrease in cell apoptosis and associated protein expression (Fig. 7D, E). Moreover, the decreased gene expression and release of proinflammatory factors were promoted by 6AN injection in Insig2-OE mouse livers subjected to IR (Fig. 7F–H). Overall, these outcomes strongly indicated that the activity of PPP is vital in mediating the protective impact of Insig2 and maintaining a balanced cellular oxidative state during liver IR injury.

### The pharmacological supply of G6P attenuates hepatic IR injury based on the Nanoparticle delivery system

**Fig. 8:**
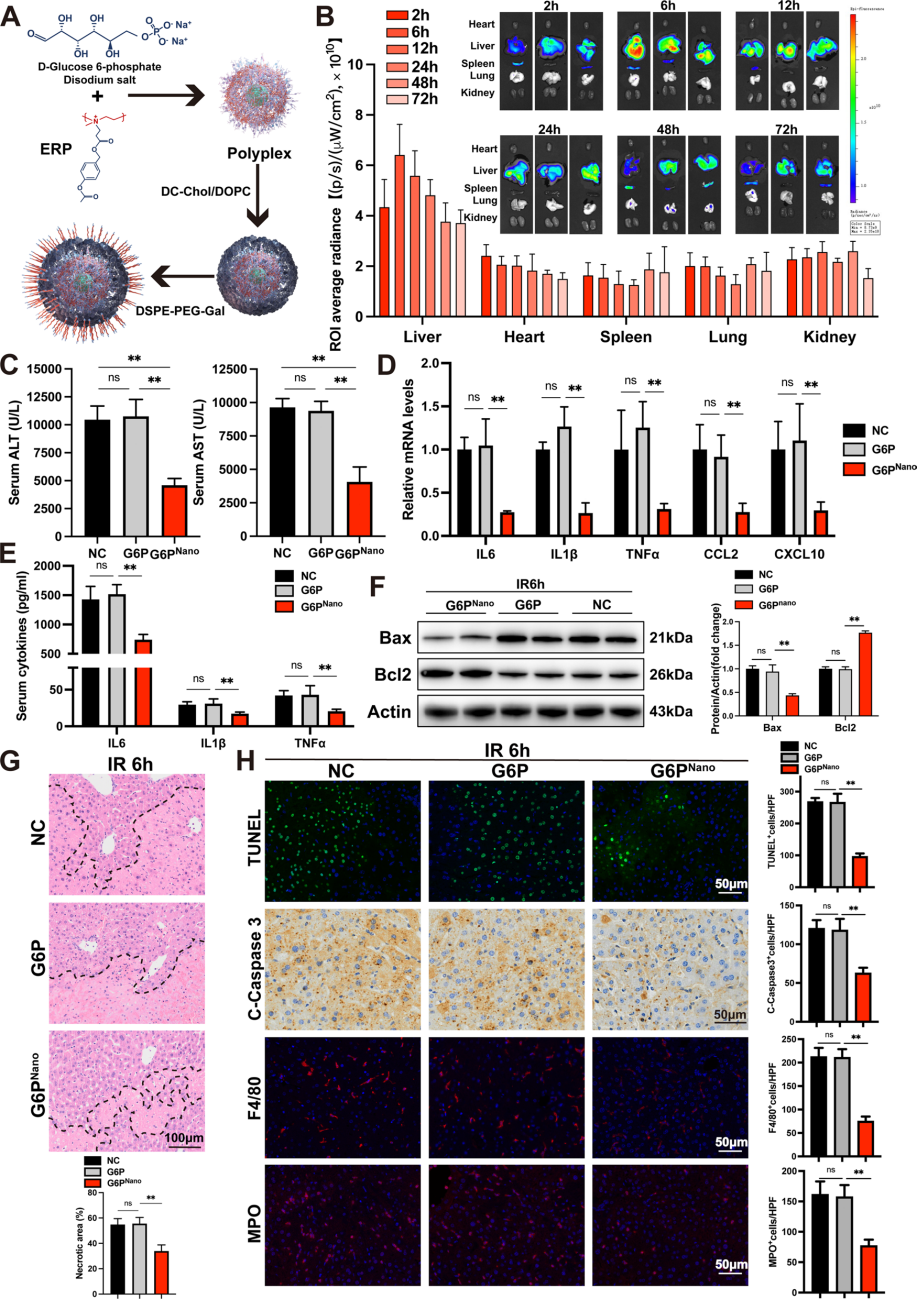
Attenuation of hepatic IR injury by liver-targeted G6P supplement by nanoparticle delivery system. **A** Chemical structure and the synthesis process of G6P nanoparticle. **B** Ex vivo bioimaging and normalized DiD-fluorescent intensity in the main organs (n = 3) of the mice IV injected with DiD-loaded PEG-Lipids/ERP/G6P (DiD, 0.75 mg/kg), and dissected and imaged at 2, 6, 12, 24, 48, or 72 h post-injection. **C** Serum levels of ALT and AST (n = 5/group). **D** Serum inflammatory cytokines (n = 5/group). **E**, **F** Hepatic mRNA levels of inflammatory factors (n = 4/group) and expression of apoptosis-related proteins (n = 2/group). **G**–**H** Liver necrosis, apoptosis and inflammatory cells enrichment in liver sections (n = 3/group). All data are shown as means ± SDs. ns indicates no significance between the two indicated groups; *p < 0.05; **p < 0.01

Finally, we discovered a metabolite––d-Glucose 6-phosphate disodium salt, a common form of glucose within the cell and could participate in the PPP and glycolysis. In vitro experiment had unveiled that AML12 cells exhibit enhanced cell viability and PPP activity upon appropriate supplementation of G6P during CoCl_2_-induced hypoxia. Furthermore, the glycolytic levels were also observed to increase (Additional file 1: Fig. S7). As a result, the subsequent phase of our study will focus on exploring the protective effects of G6P in an in vivo setting. In order to achieve the metabolite targeted-delivery to the liver, we provided a simple and safe approach for increasing G6P concentration by employing Nanoparticle delivery system (Fig. 8A). The DiD-loaded PEG-Lipids/ERP/G6P effectively accumulated in the liver and retained strong signals at 6 and 12 h post-injection. The fluorescence intensity in the liver was much stronger than other organs at all the testing times (Fig. 8B). Due to the relatively short half-life of these nanoparticles, we had chosen to administer it through two IV injections within two days, in order to maintain an effective concentration of the drug in the liver. Twenty-four hours after the first dose, the mouse liver displayed activation of PPP (Additional file 1: Fig. S8) and attenuation of the hepatic IR injury to a great extent. This was indicated by the marked suppression of biochemical liver function and proinflammatory cytokine production compared to vehicle controls (Fig. 8C–E). Histological observations showed less hepatocellular apoptosis, necrosis and recruitment of inflammation cells induced by G6P nanoparticles preconditioning compared to control mice (Fig. 8F–H).

## Discussion

Hepatic IR injury is a complex disease involving a diverse array of pathological mechanisms that induce hepatocyte death and an inflammatory response [1, 22]. Nevertheless, it remains a serious problem due to the fact that few basic research findings and therapeutic strategies have been translated into clinical practice. As a member of key molecules responding to insulin signaling in vivo, Insig2 has been extensively reported for its function in glucose and lipid metabolism. For example, it contributes to insulin's hypoglycemic effect, promotes lipid synthesis, and responds to glucagon's hypoglycemic effect [23–25]. An important function of Insig2 in hepatocytes is to regulate key enzymes involved in lipid synthesis, such as SREBP1 [26, 27]. We hypothesized that Insig2 might regulate glucolipid metabolism and energy homeostasis to influence the hepatic IR injury. In our study, the changes in expression of Insig2 observed in liver specimens from LT patients were highly consistent with those in mouse liver subjected to IR, indicating that Insig2 was closely related to hepatic IR injury. Our results showed that Insig2 deficiency aggravated hepatic IR injury, while Insig2 overexpression had the opposite effect. Primary hepatocytes were chosen to investigate whether Insig2 might affect cell apoptosis and inflammation during H/R since these cells are most abundant in the liver. Thus, our data indicated that Insig2 can attenuate hepatic IR injury in vivo and in vitro. Mechanistically, we validated that the PPP was the target of Insig2 to prevent IR injury, while the protective effect of Insig2 overexpression could be blocked by a G6PD inhibitor. Furthermore, liver-targeted delivery of PPP substrate——G6P could attenuate the IR injury in mice, which may be a potential therapeutic strategy in the future.

Hepatic IR injury consists of direct ischemic insult to hepatocytes, indirect damage resulting from the accumulation of ROS and activation of inflammatory pathways. The imbalance between the production and removal of ROS, known as OS, is a key factor in IR injury [28]. Under conditions of ischemia/hypoxia, the energy metabolism of hepatocytes shifts towards an increased glycolytic flux to satisfy their energetic requirements. When the process of reperfusion occurs, there is a significant increase in ROS formation, as oxygen and glycolytic products necessary for aerobic energy metabolism are restored. During times of excess ROS production, intrinsic antioxidant capacities become overloaded, resulting in OS [29]. In numerous studies, antioxidants have been shown promising to reduce ROS accumulations associated with IR and preserve mitochondrial function [30, 31]. Importantly, analysis of multi-omics data demonstrated that PPP was the most pronounced metabolic pathway in Insig2 overexpression liver tissue during IR. The PPP is widely known to be vital in modulating carbon homeostasis and providing precursors for nucleotide and amino acid biosynthesis [32]. Furthermore, the oxidative phase of PPP is considered the primary source of cellular NADPH, which supplies the reducing equivalents for ROS detoxification. Studies have shown that the anti-oxidative properties of PPP can protect the heart, kidney and brain against IR injury [33–36], indicating that ensuring a constant PPP activity can effectively prevent OS and maintain the equilibrium of redox homeostasis in grafts. Additionally, the non-oxidative phase of PPP supplies precursors for the biosynthesis of nucleotides and amino acids, which are essential for cell repair and regeneration.

It is generally believed that glucose is the key oxidative substrate and can be further metabolized by glycolysis or the PPP after phosphorylation to G6P by hexokinase. Therefore, any condition that activates the PPP can potentially enhance the glucose flux from glycolysis to the PPP because glycolysis and the PPP share a metabolic substrate, G6P. In our study, Insig2 deficiency significantly decreased the activity of G6PD subjected to IR at the same glucose consumption. Remarkably, this shift also involved the reduced NADPH/NADP^+^ ratios, providing fewer reducing equivalents to reduce GSSG to GSH. Additionally, we documented that the antioxidant SOD level decreased, while the oxidant MDA level increased under the condition of Insig2 deficiency subjected to IR. Given the correlation between PPP and glycolysis, our results demonstrated that Insig2 can shift the equilibrium towards PPP from glycolysis to preserve stable antioxidative PPP activity, thereby mitigating the adverse consequences of OS in the presence of IR. Notably, lactate represents the final product of glycolysis under anaerobic conditions and can be represented as a predictive biomarker for liver dysfunction [37]. Upon restoration of oxygen delivery, lactate can partially convert to pyruvate. The pyruvate can subsequently enter the Krebs cycle, undergo oxidative phosphorylation, and become a DAMP to exacerbate the inflammatory response and trigger mitochondrial ROS production [38, 39]. We discovered that the lactate content during hepatic IR declined dramatically, and hepatic lactate clearance was lower in Insig2 deficiency mice subjected to IR. Wu et al. [40] found that early systemic lactate clearance after liver transplantation was a marker of allograft function, which was consistent with our experimental results. However, there was a distinction between ischemia and reperfusion phages regarding glycolytic activity. We added CoCl_2_ to induce hypoxia, as it had been commonly utilized as a stable and reliable in vitro model in previous research studies [41]. By employing this model, we could more effectively evaluate alterations in the glycolysis and PPP in hepatocytes.

The PPP's oxidative phase is catalyzed by the first and rate-limiting enzyme G6PD and the subsequent 6-phosphogluconate dehydrogenase to produce NADPH. Competitive inhibition of G6PD with 6-AN not only accelerated liver damage subjected to IR injury, but also abolished the alteration of glucose flux towards PPP from glycolysis caused by Insig2 overexpression. These changes counteracted the protective effect of Insig2 against cell death, inflammatory response, ROS overproduction, and oxidative damage induced by hepatic IR injury. These outcomes proved that elevated PPP activity is required for Insig2 to guard the liver against IR injury.

Our outcomes supported that activation of PPP could compensate for the diminished antioxidant defenses under stress conditions, thereby functioning as a protective redox pathway. Therefore, identifying an optimal approach to stimulate PPP signaling in IR circumstances is critical. Considering the huge consumption of G6P and the interrelationship between glycolysis and PPP, we hypothesized that an appropriate supply of the substrate G6P could maintain the activity of PPP due to the apparent substrate selectively. Gluconate derivatives have been reported to prevent IR injury in the rat liver model [42]. In an in vitro hypoxia model, the viability of cells showed improvement with increasing concentrations of G6P. However, the hydrophilic nature and short half-life of the sodium salt in question pose a challenge to its effective delivery to the liver following IV administration. The advancement of organ-targeted drug delivery has resulted in the availability of novel strategies [43, 44]. Due to the high esterase expression in the liver, we utilized an amphipathic charge-reversal ERP, which can bind to hydrophilic negatively charged G6P sodium salt and coat the liposome shell through hydrophobic interactions. ERP loads G6P sodium salt through electrostatic interaction to form stable nanoparticles and coats liver-targeted long-circulating liposomes and DSPE-PEG-Gal on its surface. When injected intravenously, it rapidly targets the liver and responds to esterase hydrolysis to reverse the charge, so G6P sodium salt could play its role in protecting the liver. Based on our research findings, we concluded that G6P could participate in both the PPP and glycolysis pathways, ultimately exerting a protective role against IR injury.

This study is subject to various limitations. Initially, global KO mice were employed to investigate the role of Insig2 in hepatocytes. Further investigation is necessary to establish and validate the phenotype in liver IR injury for transgenic mice with liver-specific KO or overexpression of Insig2. Furthermore, the existing data did not investigate the precise mechanism. Therefore, more in-depth studies are required to understand better the precise mechanism behind Insig2 and PPP in liver IR injury. Additionally, we used intraventricular injection to ensure the effect of the G6P nanoparticles as a preliminary study. In research, a more advanced animal machine perfusion model should be applied.

## Conclusion

In summary, our investigation revealed that hepatocyte Insig2 is a protective factor against liver IR injury by optimizing glucose metabolism via the PPP, thus strengthening the antioxidant defenses and enhancing redox homeostasis of hepatocytes. Furthermore, we concluded that increasing the G6P levels may be a novel treatment approach for liver donor preservation.

### Supplementary Information

**Additional file 1: Figure S1.** Genotyping of WT/Insig2 KO mice. **Figure S2.** Sizes and zeta potentials of the ERP/G-6P polyplexes complexed at various mass ratios. Error bars represent the Standard deviation. **Figure S3.** Sizes and zeta potentials of ERP/G-6P polyplexes (Mass ratio = 0.1) coated with different concentrations of lipids layer and DSPC-PEG-Gal. Error bars represent the standard deviation. **Figure S4.** GO and Metascape enrichment analysis reveal after hepatic IR injury. (A-B) GO of RNA-seq data showing the most significantly enriched pathways in the livers of Insig2-OE and controlled WT mice. (C) Metascape enrichment analysis showing groups of several categories based on gene functional relevance, and construction of a network was based on relevance and similarity. In the figure, different colors are used to represent different categories. (D-E) GO of proteomics data showing the most significantly enriched pathways. (F) The functional enrichment analysis of DEPs using Metascape. **Figure S5.** Transcriptome, proteome, and metabolome enrichment of the PPP in Insig2-OE and controlled WT groups. (A) Pathway analysis of DEGs, DEPs, DEMs were performed with the KEGG. (B-C) Heat map showing genes (A) and proteins (B) in the “PPP” metabolic pathway. (D). Changes in PPP metabolites. **Figure S6.** Inhibition of PPP mediates redox imbalance and the protective impact of Insig2. (A-E) The G6PD activity (n = 3/group), NADPH/NADP + ratio (n = 3/group), GSH/GSSG ratio (n = 3/group), SOD activity (n = 3/group), MDA level (n = 3/group) in liver tissues were detected at IR group from Insig2-OE and controlled WT mice with 6-AN or DMSO. **Figure S7.** G6P protects AML12 cells from CoCl2-induced hypoxic injury. (A) The AML12 cells were subjected to the CCK-8 assay to detect the effective concentration of G6P disodium salt. (B-D) The G6PD activity (n = 4/group), NADPH/NADP + ratio (n = 4/group) and measurement of ECAR in AML12 cells incubated with G6P disodium salt. **Figure S8.** The Characterization and in vivo effect of nanoparticles. (A) Size distribution of G6P nanoparticle. (B) The G6PD activity (n = 4/group), NADPH/NADP + ratio (n = 4/group) in IR liver tissues injected with G6P or G6P nanoparticles. **Table S1.** Primers for real-time qPCR detection. **Table S2.** Antibodies for immunoblot analyses.

## Data Availability

The datasets used and/or analyzed during the current study are available from the corresponding author on reasonable request.

## Abbreviations

Insig2: Insulin-induced gene 2
IR: Ischemia–reperfusion
LT: Liver transplantation
KO: Knockout
H/R: Hypoxia/reoxygenation
AAV8: Adeno-associated-virus 8
CoCl_2_: Cobalt chloride
PPP: Pentose phosphate pathway
G6PD: Glucose-6-phosphate-dehydrogenase
G6P: Glucose 6-phosphate
ROS: Reactive oxygen species
DAMPs: Damage-associated molecular patterns
ER: Endoplasmic reticulum
Insig: Insulin-induced gene
SREBP: Sterol regulatory element-binding protein
SCAP: SREBP cleavage-activating protein
PCR: Polymerase chain reaction
ALT: Alanine aminotransferase
AST: Aspartate aminotransferase
ELISA: Enzyme-linked immunosorbent assay
H&E: Hematoxylin and eosin
IHC: Immunohistochemistry
TUNEL: Terminal deoxynucleotidyl transferase-mediated deoxyguanosine triphosphate nick-end labeling
NADPH/NADP^+^: Reduced nicotinamide adenine dinucleotide phosphate/nicotinamide adenine dinucleotide phosphate
OS: Oxidative stress
GSH/GSSG: Glutathione/glutathione disulfide
SOD: Superoxide Dismutase
MDA: Malondialdehyde
DMEM: Dulbecco’s modified Eagle’s medium
FBS: Fetal bovine serum
TBG: Thyroxine-binding globulin
shRNA: Short-hairpin RNA
siRNA: Small interference RNA
DEGs: Differentially expressed genes
KEGG: Kyoto Encyclopedia of Genes and Genomes
GO: GeneOntology
DEPs: Differentially expressed proteins
DEMs: Differentially abundant metabolites
OPLS-DA: Orthogonal projections to latent structure-discriminant analysis
VIP: Variable importance in projection
ECAR: Extracellular acidification rate
ERP: Esterase-responsive polymer
IV: Intravenous
ANOVA: One-way analysis of variance
OLT: Orthotopic liver transplantation
sALT: Serum ALT
WT: Wild-type
Bcl-2: B-cell leukemia/lymphoma
Bax: Bcl2-associated X protein
Ccl2: C–C motif chemokine ligand 2
Cxcl10: CXC motif chemokine ligand 10
Insig2-OE: Insig2-overexpression
GFP: Green fluorescent protein
6-AN: 6-Aminonicotinamide
