# Supplementary material for: Insulin-induced gene 2 protects against hepatic ischemia–reperfusion injury via metabolic remodeling
Source: J Transl Med. 2023 Oct 19;21:739. doi: 10.1186/s12967-023-04564-y (PMC10585752; doi:10.1186/s12967-023-04564-y)
Supplement: Supplementary file 1 — Additional file 1: Figure S1. Genotyping of WT/Insig2 KO mice. Figure S2. Sizes and zeta potentials of the ERP/G-6P polyplexes complexed at various mass ratios. Error bars represent the Standard deviation. Figure S3. Sizes and zeta potentials of ERP/G-6P polyplexes (Mass ratio = 0.1) coated with different concentrations of lipids layer and DSPC-PEG-Gal. Error bars represent the standard deviation. Figure S4. GO and Metascape enrichment analysis reveal after hepatic IR injury. (A-B) GO of RNA-seq data showing the most significantly enriched pathways in the livers of Insig2-OE and controlled WT mice. (C) Metascape enrichment analysis showing groups of several categories based on gene functional relevance, and construction of a network was based on relevance and similarity. In the figure, different colors are used to represent different categories. (D-E) GO of proteomics data showing the most significantly enriched pathways. (F) The functional enrichment analysis of DEPs using Metascape. Figure S5. Transcriptome, proteome, and metabolome enrichment of the PPP in Insig2-OE and controlled WT groups. (A) Pathway analysis of DEGs, DEPs, DEMs were performed with the KEGG. (B-C) Heat map showing genes (A) and proteins (B) in the “PPP” metabolic pathway. (D). Changes in PPP metabolites. Figure S6. Inhibition of PPP mediates redox imbalance and the protective impact of Insig2. (A-E) The G6PD activity (n = 3/group), NADPH/NADP + ratio (n = 3/group), GSH/GSSG ratio (n = 3/group), SOD activity (n = 3/group), MDA level (n = 3/group) in liver tissues were detected at IR group from Insig2-OE and controlled WT mice with 6-AN or DMSO. Figure S7. G6P protects AML12 cells from CoCl2-induced hypoxic injury. (A) The AML12 cells were subjected to the CCK-8 assay to detect the effective concentration of G6P disodium salt. (B-D) The G6PD activity (n = 4/group), NADPH/NADP + ratio (n = 4/group) and measurement of ECAR in AML12 cells incubated with G6P disodium salt. Figure S8. The Characteriz [file 12967_2023_4564_MOESM1_ESM.docx]

**Additional file 1**

**Additional Figures**

**Figure S1. Genotyping of WT/Insig2 KO mice.**

**Figure S2. sizes and zeta potentials of the ERP/G-6P polyplexes complexed at various mass ratios. Error bars represent the Standard deviation.**

**Figure S3.** **Sizes and zeta potentials of** **ERP/G-6P polyplexes (Mass ratio=0.1) coated with different concentrations of lipids layer and DSPC-PEG-Gal.** **Error bars represent the standard deviation.**

**Figure S4.** **GO and Metascape enrichment analysis reveal after hepatic IR injury.** (A-B) GO of RNA-seq data showing the most significantly enriched pathways in the livers of Insig2-OE and controlled WT mice. (C) Metascape enrichment analysis showing groups of several categories based on gene functional relevance, and construction of a network was based on relevance and similarity. In the figure, different colors are used to represent different categories. (D-E) GO of proteomics data showing the most significantly enriched pathways. (F) The functional enrichment analysis of DEPs using Metascape.

**Figure S5. Transcriptome, proteome, and metabolome enrichment of the PPP in Insig2-OE and controlled WT groups.** (A) Pathway analysis of DEGs, DEPs, DEMs were performed with the KEGG. (B-D) Heat map showing genes (B), proteins (C) and metabolites (D) in the “PPP” metabolic pathway.

**Figure S6. Inhibition of PPP mediates redox imbalance and the protective impact of Insig2.** (A-E) The G6PD activity (n=3/group), NADPH/NADP^+^ ratio (n=3/group), GSH/GSSG ratio (n=3/group), SOD activity (n=3/group), MDA level (n=3/group) in liver tissues were detected at IR group from Insig2-OE and controlled WT mice with 6-AN or DMSO.

**Figure S7. G6P protects AML12 cells from CoCl_2_-induced hypoxic injury.** (A) The AML12 cells were subjected to the CCK-8 assay to detect the effective concentration of G6P disodium salt. (B-D) The G6PD activity (n=4/group), NADPH/NADP^+^ ratio (n=4/group) and measurement of ECAR in AML12 cells incubated with G6P disodium salt.

**Figure S8. The Characterization and in vivo effect of nanoparticles.** (A) Size distribution of G6P nanoparticle. (B) The G6PD activity (n=4/group), NADPH/NADP^+^ ratio (n=4/group) in IR liver tissues injected with G6P or G6P nanoparticles.

**Additional Tables**

**Table S1. Primers for real-time qPCR detection**

| **Gene** |  | **Sequence5'---3'** |
| --- | --- | --- |
| **Mouse Insig2** | **F** | GGAGTCACCTCGGCCTAAAAA |
|  | **R** | CAAGTTCAACACTAATGCCAGGA |
| **Mouse IL6** | **F** | CTGCAAGAGACTTCCATCCAG |
|  | **R** | AGTGGTATAGACAGGTCTGTTGG |
| **Mouse IL1β** | **F** | TTCAGGCAGGCAGTATCACTC |
|  | **R** | GAAGGTCCACGGGAAAGACAC |
| **Mouse TNF-α** | **F** | CAGGCGGTGCCTATGTCTC |
|  | **R** | CGATCACCCCGAAGTTCAGTAG |
| **Mouse Ccl2** | **F** | TAAAAACCTGGATCGGAACCAAA |
|  | **R** | GCATTAGCTTCAGATTTACGGGT |
| **Mouse Cxcl10** | **F** | CCAAGTGCTGCCGTCATTTTC |
|  | **R** | GGCTCGCAGGGATGATTTCAA |
| **Mouse Bax** | **F** | AGACAGGGGCCTTTTTGCTAC |
|  | **R** | AATTCGCCGGAGACACTCG |
| **Mouse Bcl2** | **F** | GCTACCGTCGTGACTTCGC |
|  | **R** | CCCCACCGAACTCAAAGAAGG |
| **Mouse** **β-actin** | **F** | GTGACGTTGACATCCGTAAAGA |
|  | **R** | GCCGGACTCATCGTACTCC |

**Table S2. Antibodies for immunoblot analyses**

| **Antibody** | **Cat No.** | **Manufacture** |
| --- | --- | --- |
| **Insig2** | 24766-1-AP | Proteintech |
| **Bax** | 2772S | CST |
| **Bcl2** | ab182858 | Abcam |
| **β-actin** | 66009-1-Ig | Proteintech |
